# Plants Pre-Infested With Viruliferous MED/Q Cryptic Species Promotes Subsequent *Bemisia tabaci* Infestation

**DOI:** 10.3389/fmicb.2018.01404

**Published:** 2018-06-27

**Authors:** Xiaobin Shi, Gong Chen, Huipeng Pan, Wen Xie, Qingjun Wu, Shaoli Wang, Yong Liu, Xuguo Zhou, Youjun Zhang

**Affiliations:** ^1^Hunan Academy of Agricultural Sciences, Hunan Plant Protection Institute, Changsha, China; ^2^Institute of Vegetables and Flowers, Chinese Academy of Agricultural Sciences, Beijing, China; ^3^College of Plant Protection, Hunan Agricultural University, Changsha, China; ^4^Department of Entomology, University of Kentucky, Lexington, KY, United States

**Keywords:** *Bemisia tabaci*, plant volatile, neophytadiene, thujene, ρ-cymene, tomato yellow leaf curl virus

## Abstract

The sweet potato whitefly, *Bemisia tabaci*, is one of the most invasive insect pests worldwide. The two most destructive whitefly cryptic species are MEAM1/B and MED/Q. Given that MED/Q has replaced MEAM1/B in China and the invasion of MED/Q has coincided with the outbreak of tomato yellow leaf curl virus (TYLCV), we hypothesize that pre-infestation with viruliferous *B. tabaci* will affect the subsequent host preferences. To test this hypothesis, we (1) conducted bioassays to compare the host preference of viruliferous and non-viruliferous MEAM1/B and MED/Q, respectively, on plants pre-infested with viruliferous and non-viruliferous MEAM1/B and MED/Q; (2) profiled plant volatiles using GC-MS; and (3) functionally characterized chemical cues could potentially modulate *B. tabaci*-TYLCV-tomato interactions, including ρ-cymene, thujene and neophytadiene, using a Y-tube olfactometer. As a result, plants pre-infested with MEAM1/B whiteflies carrying TYLCV or not, did not attract more or less B or Q whiteflies. Plants pre-infested with non-viruliferous MED/Q resisted MEAM1/B but did not affect MED/Q. However, plants pre-infested with viruliferous MED/Q attracted more whiteflies. Feeding of viruliferous MED/Q reduced the production of ρ-cymene, and induced thujene and neophytadiene. Functionally analyses of these plant volatiles show that ρ-cymene deters while neophytadiene recruits whiteflies. These combined results suggest that pre-infestation with viruliferous MED/Q promotes the subsequent whitefly infestation and induces plant volatile neophytadiene which recruits whiteflies.

## Introduction

Plant virus spread and transmission is, at least partially, dependent on the virus–vector interaction, which can be modified by the virus to obtain an adaptive advantage (Blua and Perring, 1992). A better understanding of the mechanisms mediating such relationship is important to reveal the interaction ecology and vector–virus co-evolution, and may serve as a basis for manipulating vectors to limit virus spread in plants.

*Bemisia tabaci* (Gennadius) (Hemiptera: Aleyrodidae) is a phloem-feeding invasive pest that causes severe damages in vegetables and crops. *B. tabaci* MEAM1/B and MED/Q are the two most invasive whiteflies worldwide (Perring et al., 2001; Jones, 2003; De Barro et al., 2011). Most recently, MED/Q has replaced MEAM1/B, and became the dominant *B. tabaci* cryptic species in China (Pan et al., 2012; Liu et al., 2013). Tomato yellow leaf curl virus (TYLCV), genus *Begomovirus*, family *Geminiviridae*, a serious threat to tomato production globally, is transmitted exclusively by *B. tabaci* in a persistent and circulative manner (Czosnek and Laterrot, 1997; Moriones and Navas-Castillo, 2000; Navas-Castillo et al., 2011). TYLCV outbreak coincided with MED/Q invasion in China, and previous studies have shown that TYLCV infection differentially changed the life history and feeding behavior of MEAM1/B and MED/Q, respectively (Pan et al., 2011, 2012; Liu et al., 2013). Chemosensory proteins (CSPs) has been shown to be involved in different insecticide resistance capacity of MEAM1/B and MED/Q, and MED/Q apparently contains more CSP genes than MEAM1/B (Liu et al., 2016).

Within plant-virus-insect tritrophic interactions, both insects and plants emit and receive chemical cues that modulate their interactions (Howe and Jander, 2008). Feeding behavior of whiteflies is affected by plant odors, and to exploit natural volatile signaling processes is a new strategy to manipulate whitefly behavior (Schlaeger et al., 2018). Besides, odorant binding proteins (OBPs) and CSPs of whiteflies can be effective targets for pest control as they may be involved in mating choice and host location of *B. tabaci* (Wang et al., 2017). However, there are still many gaps in whiteflies and odors research.

Infection by persistently transmitted virus can change plant volatile profile and the subsequent responses of its vector (Eigenbrode et al., 2002; Alvarez et al., 2007). Similarly, feeding can also affect the subsequent herbivore behavior and performance because of the defensive signaling and responses from plants (Sauge et al., 2006; Poelman et al., 2008; Sarmento et al., 2011a,b). Given the fact that MED/Q replaced MEAM1/B in China and the outbreak of virus is associated with the invasion of MED/Q, we hypothesize that *B. tabaci* will respond differently toward plants previously infested with viruliferous MEAM1/B and viruliferous MED/Q, respectively.

To test this hypothesis, we (1) conducted bioassays to compare the host preference of *B. tabaci* MEAM1/B and MED/Q on plants previously infested with viruliferous and non-viruliferous MEAM1/B and MED/Q, respectively; (2) profiled plant volatiles using GC-MS; and (3) functionally characterized ρ-cymene, thujene and neophytadiene, chemical cues that potentially modulate *B. tabaci*-TYLCV-tomato interactions using a Y-tube olfactometer.

## Results

### Preference of *B. tabaci* on Plants Previously Infested With Non-viruliferous or Viruliferous MEAM1/B and MED/Q

**FIGURE 1:**
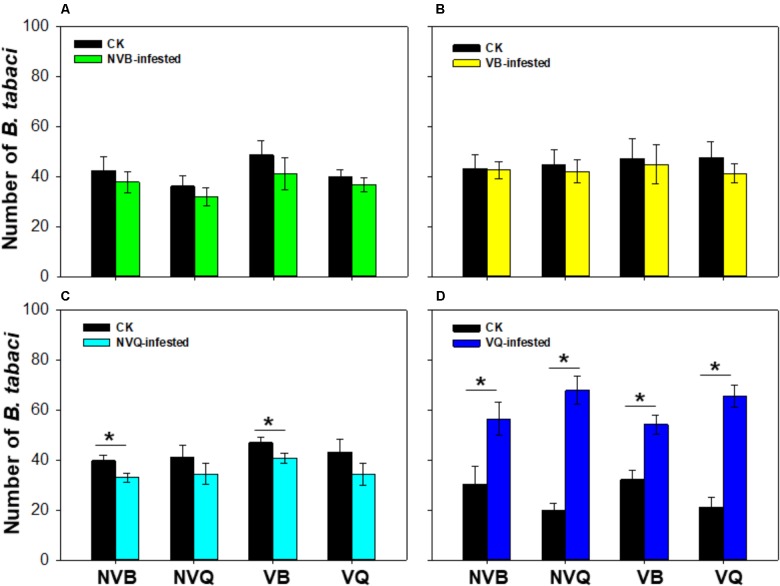
Preference of *B. tabaci* on plants previously exposed to non-viruliferous and viruliferous MEAM1/B and MED/Q. **(A)** Preference of *B. tabaci* on plants previously infested with non-viruliferous MEAM1/B. **(B)** Preference of *B. tabaci* on plants previously infested with viruliferous MEAM1/B. **(C)** Preference of *B. tabaci* on plants previously infested with non-viruliferous MED/Q. **(D)** Preference of *B. tabaci* on plants previously infested with viruliferous MED/Q. CK: Plants were non-infested. NVB-infested: Plants were infested with non-viruliferous MEAM1/B. NVQ-infested: Plants were infested with non-viruliferous MED/Q. VB-infested: Plants were infested with viruliferous MEAM1/B. VQ-infested: Plants were infested with viruliferous MED/Q. Values are means ± SE (*n* = 9). Asterisks indicate significant differences (*P* < 0.05) between treatments by *t*-test.

The number of *B. tabaci* was similar on plants that were previously infested with non-viruliferous MEAM1/B and on undamaged plants (*P* = 0.404; **Figure 1A**). Similarly, the number of *B. tabaci* showed no difference on plants that were previously infested with viruliferous MEAM1/B and on undamaged plants (*P* = 0.652; **Figure 1B**). The number of non-viruliferous MEAM1/B and viruliferous MEAM1/B was significantly lower on non-viruliferous MED/Q -infested plants than on undamaged plants (*P* = 0.006; **Figure 1C**). However, the number of *B. tabaci* was significantly higher on viruliferous MED/Q -damaged plants than on undamaged plants (*P* < 0.001; **Figure 1D**).

### Extraction and Analysis of Plant Volatiles

**FIGURE 2:**
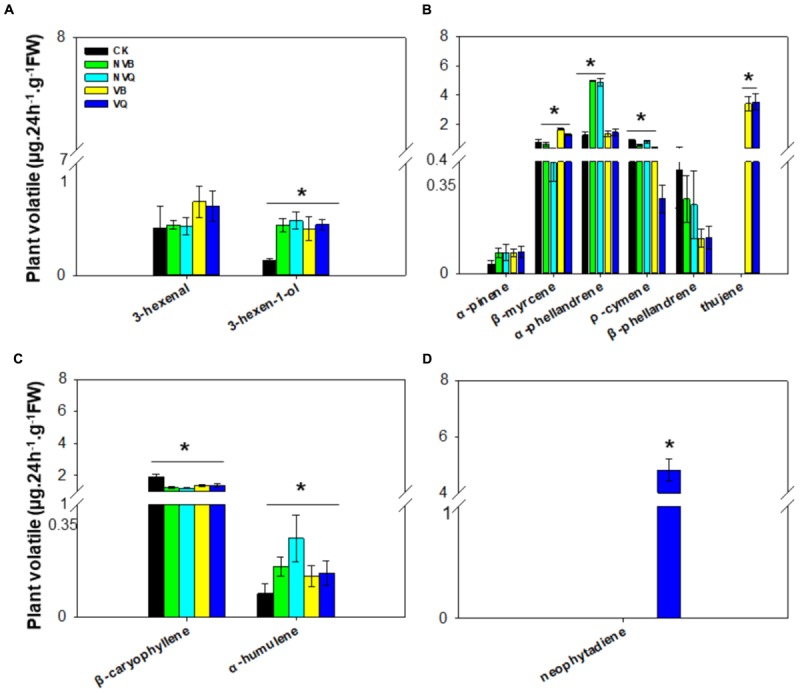
Volatiles generated from non-infested and infested host plants with non-viruliferous and viruliferous MEAM1/B and MED/Q. **(A)** Aliphatic compound (in the order of 3-hexenal and 3-hexen-1-ol); **(B)** Monoterpene (in the order of α-pinene, β-myrcene, α-phellandrene; ρ-cymene; β-phellandrene and thujene); **(C)** Sesquiterpene (in the order of β-caryophyllene and α-humulene); **(D)** Diterpene (neophytadiene). CK: Plants were non-infested. NVB: Plants were infested with non-viruliferous MEAM1/B. NVQ: Plants were infested with non-viruliferous MED/Q. VB: Plants were infested with viruliferous MEAM1/B. VQ: Plants were infested with viruliferous MED/Q. All plants were infested with 300 *B. tabaci* 2 days before volatile trapping. Values are means ± SE (*n* = 9). Asterisks indicate significant differences (*P* < 0.05) between the control volatiles and whitefly-infested volatiles by Tukey’s HSD test.

A total of four classes of plant volatiles, including aliphatic compound, monoterpenes, sesquiterpenes and diterpene, were detected. The content of 3-hexen-1-ol, which belongs to aliphatic compound, differed in plants infested and non-infested, but did not differ in plants infested with non-viruliferous and viruliferous MEAM1/B and MED/Q (**Figure 2A**). Among monoterpenes, β-myrcene, α-phellandrene, ρ-cymene and thujene differed in all five treatments (**Figure 2B**). The ρ-cymene was reduced on viruliferous MED/Q -infested plants compared to control plants and plants infested with other whiteflies. Thujene was induced exclusively on plants infested with viruliferous MEAM1/B and MED/Q. The content of sesquiterpenes, such as β-caryophyllene and α-humulene, also differed in all five treatments (**Figure 2C**). Neophytadiene was only detected on viruliferous MED/Q-damaged plants (**Figure 2D**).

### Functionally Characterization of ρ-Cymene, Thujene and Neophytadiene

**FIGURE 3:**
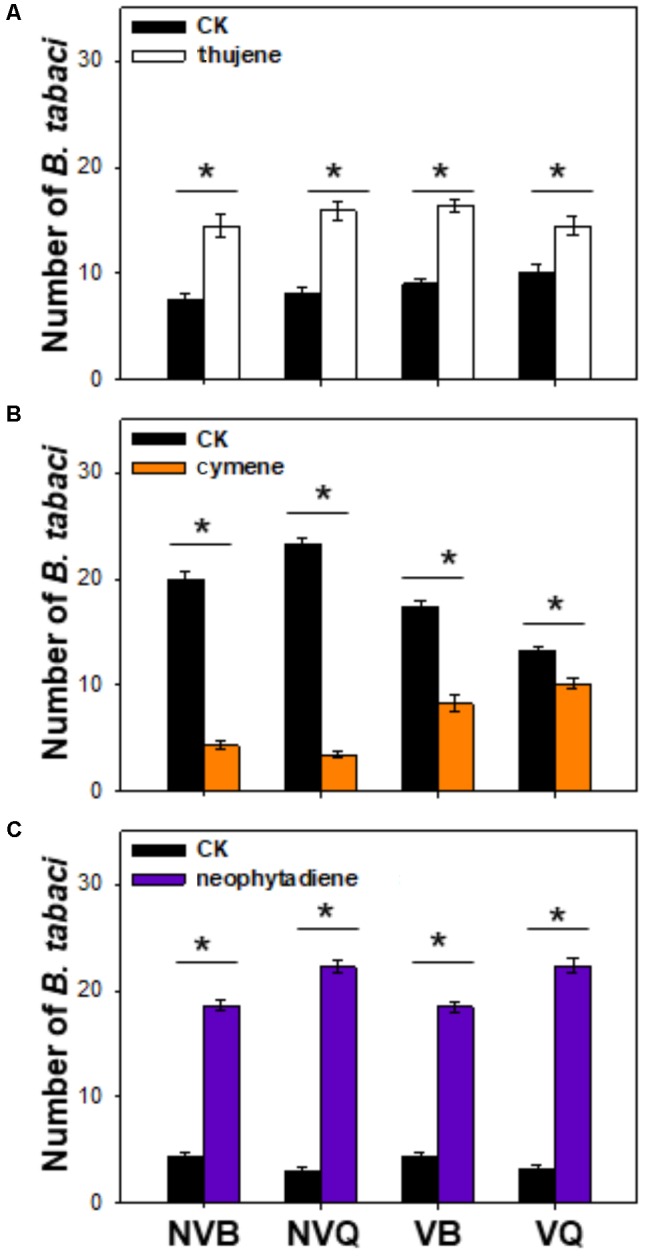
Preference of *B. tabaci* for selected plant volatiles. Preference of *B. tabaci* for thujene **(A)**, ρ-cymene **(B)**, and neophytadiene **(C)** was investigated using a Y-tube olfactometer. Values are means ± SE (*n* = 9). Asterisks indicate significant differences (*P* < 0.05) between the control and the tested volatiles by a general linear model.

Thujene attracted significantly more *B. tabaci* in comparison to the control (purified air), while the number of non-viruliferous and viruliferous MEAM1/B and MED/Q showed no differences (**Figure 3A**). Neophytadiene also attracted *B. tabaci*, especially for MED/Q, regardless of the previous treatment (**Figure 3B**). ρ-cymene, on the other hand, repelled *B. tabaci*, especially for non-viruliferous MEAM1/B and non-viruliferous MED/Q (**Figure 3C**).

**FIGURE 4:**
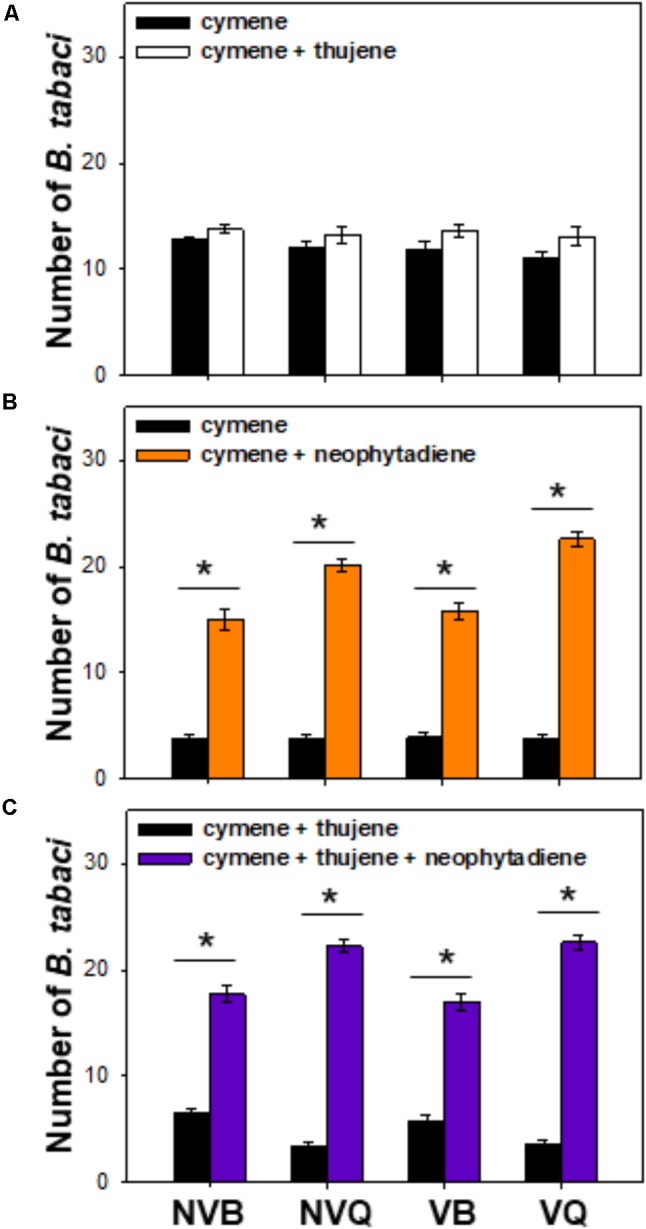
Preference of *B. tabaci* for plant volatiles with different combinations. Preference of *B. tabaci* for ρ-cymene vs. ρ-cymene + thujene **(A)**, ρ-cymene vs. ρ-cymene + neophytadiene **(B)**, and ρ-cymene + thujene vs. ρ-cymene + thujene + neophytadiene **(C)** was investigated using a Y-tube olfactometer. Values are means ± SE (*n* = 9). Asterisks indicate significant differences (*P* < 0.05) between two treatments by a general linear model.

Compared to ρ-cymene, a mixture of thujene and ρ-cymene showed no effect on *B. tabaci* (**Figure 4A**). *B. tabaci*, especially non-viruliferous and viruliferous MED/Q, showed a much higher preference to a mixture of ρ-cymene and neophytadiene than to ρ-cymene (**Figure 4B**). Compared to a mixture of ρ-cymene and thujene, a blend of ρ-cymene, thujene and neophytadiene attracted more *B. tabaci*, especially for MED/Q (either viruliferous or not) (**Figure 4C**).

## Discussion

Plants with previous exposures to MEAM1/B, non-viruliferous or viruliferous, did not affect the preference of the subsequent *B. tabaci*. However, plants pre-infested with MED/Q, non-viruliferous or viruliferous, indeed changed the host preference of the subsequent feeders. Plants pre-infested with non-viruliferous MED/Q repelled the subsequent feeding of MEAM1/B; while pre-infestation by viruliferous MED/Q attracted both MEAM1/B and MED/Q. Similar to semiochemicals, plant volatiles can attract or repel insects (Moraes et al., 1998; Poecke and Dicke, 2002; Kappers et al., 2005; Blanc and Michalakis, 2016). In this research, *B. tabaci* responded differently to plants pre-infested with non-viruliferous and viruliferous MEAM1/B and MED/Q. Plant volatiles, induced by *B. tabaci* feeding, exhibited a different chemical profile, based on the presence or absence of virus in the vector insects.

Monoterpenes, including β-myrcene, α-phellandrene, ρ-cymene and thujene, were differentially induced during non-viruliferous or viruliferous MEAM1/B and MED/Q infestation. In comparison to control plants and plants pre-infested with non-viruliferous *B. tabaci*, ρ-cymene was significantly reduced on plants pre-infested with viruliferous MED/Q. ρ-cymene has been shown to exhibit repellent properties in tomato-whitefly interactions (Bleeker et al., 2009; Fang et al., 2013), which is consistent with our current result. These results indicate that the reduction of ρ-cymene may contribute to the preference of viruliferous MED/Q -infested plants.

As for the terpenoids, thujene was induced exclusively on plants pre-infested with viruliferous MEAM1/B and MED/Q. Similarly, neophytadiene was induced only on plants pre-infested with viruliferous MED/Q. Individually tested, thujene and neophytadiene were attractive to *B. tabaci*, especially MED/Q. When mixed with ρ-cymene, either thujene or neophytadiene, increased the number of whiteflies. In addition, *B. tabaci* was attracted to a mixture of ρ-cymene, thujene, and neophytadiene.

Plant volatiles play important roles in push-pull technology which combines repellents and attractants in the same cropping system (Schlaeger et al., 2018). According to our results, whiteflies were deterred from non-viruliferous MED/Q-infested plants and lured to plants infested with viruliferous MED/Q at the same time. The volatiles ρ-cymene and neophytadiene may play important roles in this process. OBPs can be involved in chemical recognition which regulates host choice of whiteflies (Wang et al., 2017), and more information about the olfactory system of whiteflies is needed. Besides, field studies are also needed to confirm the push and pull role of the volatiles ρ-cymene and neophytadiene.

The recruitment function of neophytadiene, which is induced solely by viruliferous MED/Q, gives MED/Q an advantage for the subsequent feeding. Previously, we found that feeding of viruliferous MED/Q reduced the jasmonic acid (JA) content, therefore *B. tabaci* performed better on viruliferous MED/Q-infested plants (Shi et al., 2014). Our latest research suggests that the synthesis of JA and neophytadiene is antagonistic, and the titer of neophytadiene is higher in low- than in high-JA plants (unpublished data). We suspect that a specific protein within JA pathway may inhibit the biosynthesis of neophytadiene. While feeding by viruliferous MED/Q reduces the synthesis of JA, it increases the production of neophytadiene.

Such tritrophic interaction between plant, insect vectors and virus eventually facilitates virus transmission. For example, cucumber mosaic virus (CMV) employs a 2b protein to target host’s JA pathway to manipulate host’s attractiveness to insect vectors (Ziebell et al., 2011; Wu et al., 2017). The viral satellite-coded βC1 protein of tomato yellow leaf curl China virus (TYLCCNV) interacts with the host protein AS1 to suppress JA-related responses to enhance the fitness of whiteflies (Zhang et al., 2012). Besides, βC1 protein also interacts with MYC2, a key component within the JA pathway, to activate terpene synthase and to establish a mutualistic relationship with *B. tabaci* (Li et al., 2014). The NIa-Pro protein of turnip mosaic virus (TuMV) manipulates the physiology of host plants to attract aphids to promote their reproduction (Casteel et al., 2014). In addition, the ethylene signaling is modified in the NIa-Pro-associated interference in plant defense; as a result, it facilitates the virus transmission by aphids (Casteel et al., 2015). TYLCV is a true monopartite begomovirus that lacks a beta-satellite (Zhou, 2013), and the protein of TYLCV interacting with the JA pathway may be different from TYLCCNV. Therefore, for the whitefly management purposes, further research focusing on the key protein(s) within the JA synthesis pathway, which can reduce neophytadiene production, is warranted.

Herbivores and pathogens have evolved counter-adaptations to manipulate direct and indirect plant defenses (Alba et al., 2011). In China, MED/Q not MEAM1/B is associated with the transmission of TYLCV. Previous research shows that insecticide resistance is one of the driving forces in the replacement of MEAM1/B by MED/Q (Pan et al., 2015). Some CSPs of MEAM1/B and MED/Q of whiteflies are crucial to facilitate the transport of fatty acids thus develop different resistance to insecticides (Liu et al., 2016). Based on our results, post-infection volatile changes is likely the other key factor contributing to the endemic feature of TYLCV in China.

Within plant-virus-insect tritrophic interactions, both insects and plants emit and receive chemical cues that modulate their interactions (Howe and Jander, 2008). Feeding behavior of whiteflies is affected by plant odors, and to exploit natural volatile signaling processes is a new strategy to manipulate whitefly behavior (Schlaeger et al., 2018). Besides, however, there are still many gaps in whiteflies and odors research.

## Materials and Methods

### Host Plants

Tomato plants (*Solanum lycopersicum* Mill. cv. Zhongza 9) were grown in a glasshouse for this experiment. TYLCV**-**infected tomato plants were produced at the 3**-**4 true leaf stage by *Agrobacterium tumefaciens***-**mediated inoculation with a cloned TYLCV genome (GenBank accession ID: AM282874), which was originally isolated from Shanghai, China (Wu et al., 2006). Infection was carried out when the optical density (OD 600) of *A. tumefaciens* solution reached between 0.6 and 0.8. Viral infection of test plants was confirmed by the development of characteristic leaf curl symptoms and by molecular analysis with the TYLCV primer set TYLCV-61 and TYLCV-473 (Pan et al., 2012).

### Establishment of Non-viruliferous and Viruliferous *B. tabaci* Colonies

*Bemisia tabaci* MEAM1/B were originally collected from an infested cabbage (*Brassica oleracea*. cv. Jingfeng 1) in a field in Beijing, China in 2004, and *B. tabaci* MED/Q were originally collected from infested poinsettia (*Euphorbia pulcherrima* Wild. ex Klotz.) in Beijing, China in 2009 (Pan et al., 2012). We created four whitefly colonies: non-viruliferous MEAM1/B, non-viruliferous MED/Q, viruliferous MEAM1/B, and viruliferous MED/Q. The rearing of whitefly colonies and determination of whitefly purity followed the protocol described previously (Shi et al., 2013).

### Host Preference Bioassay

Tomato plants were infested with 300 viruliferous or non-viruliferous MEAM1/B or MED/Q for 2 days. After this period, all whiteflies were removed, and all of the previously infested plants and noninfested control plants were transferred to new whitefly-proof screen cages (80 cm × 40 cm × 60 cm), with two plants (one previously infested with whiteflies and one control) per cage. The two plants were placed 40 cm apart in opposite corners of the cage. About 100 adult whiteflies, which had been starved for 24 h, were collected at 7:00 am with aspirating equipment and released into the center of the screen cage above the plant. The number of whiteflies on each previously infested or control plant was determined after 6 h. To avoid whiteflies escaping and relocating during counting, whiteflies with plants were covered in transparent plastic fresh package respectively under the dim light. The open end of the package was tied to the plant stalks with a rope. In each package, the number of whiteflies on plant was counted under the bright light. Within each pair of infested and control plants, preference of non-viruliferous MEAM1/B, non-viruliferous MED/Q, viruliferous MEAM1/B, and viruliferous MED/Q were determined. The preference experiment was repeated nine times, including each pair of nine previously infested plants and nine non-infested control plants. In total, there were 36 plants previously infested with non-viruliferous MEAM1/B, non-viruliferous MED/Q, viruliferous MEAM1/B, and viruliferous MED/Q, and 144 noninfested control plants.

### Extraction and Analysis of Volatiles

Tomato plants were infested with 300 viruliferous or non-viruliferous MEAM1/B or MED/Q whiteflies for 2 days. Each treatment was repeated nine times, including nine plants infested with 300 non-viruliferous MEAM1/B, non-viruliferous MED/Q, viruliferous MEAM1/B, or viruliferous MED/Q, and nine noninfested control plants. There were 45 plants in total. After 2 days, plant volatiles emitted by whiteflies-infested or noninfested control tomato plants were collected using a headspace collection system as described previously with minor modification (Zhang et al., 2013). After 6 h of trapping under continuous light, the plants were weighed (FW).

Headspace samples were dissolved in n-hexane and 0.2 μg⋅mL^−1^ of n-dodecane was added to the solvent as an internal standard. Then a 1 μL sample of the supernatant was subjected to gas chromatography–mass spectrometry. The temperature profile was as follows: 50°C 1 min; 50°C to 240°C, 5°C min^−1^; 240°C 2 min; 240°C to 300°C, 30°C min^−1^; 300°C 5 min. The injection temperature was 270°C. The temperature of the source was 200°C, and the interface temperature was 280°C. The column effluent was ionized by electron impact ionization (70 eV).

Compounds were identified by comparison of GC retention times and normalization of peak areas with those of internal standard and true standards whenever possible and by comparison of mass spectra with spectra of the National Institute of Standards and Technology (NIST) database. When standards were not available, the concentrations of the compounds were matched to published information (Ament et al., 2004; Kant et al., 2004).

### Y-Tube Olfactometer Tests

Preferences of whiteflies to plant volatiles were determined in a Y-tube olfactometer. According to the plant volatile results, three standards of ρ-cymene, thujene and neophytadiene were used to test the preference of whiteflies. Two streams of purified air (filtered through activated charcoal) were led through two glass containers (one of standard chemical and one of purified air as control) into the olfactometer arms at 100 ml min^−1^. The experiment started with the release of individual female adult whitefly at the base of the Y-tube. Each whitefly was observed for a maximum of 20 min. A ‘no choice’ was recorded when the adults remained inactive during the testing period, and a choice for one of the two odor sources was recorded when the whitefly moved >5 cm onto either arm and stayed in that arm for at least 15 s. There were four whitefly populations for each odor comparison: non-viruliferous MEAM1/B, viruliferous MEAM1/B, non-viruliferous MED/Q and non-viruliferous MED/Q. For each population, preference of 30 whiteflies was tested for three times each day. Then the experiment was repeated for 3 days. That is to say, a total of 270 whiteflies in each population were tested for each odor comparison.

### Statistical Analysis

All proportional data were arcsine-square root transformed before analyses. Preference of viruliferous and non-viruliferous MED/Q and MEAM1/B on plants previously exposed to viruliferous and non-viruliferous MEAM1/B and MED/Q were compared with *t*-tests. One-way ANOVA was used to compare the volatile released on uninfested plants and on plants infested with non-viruliferous and viruliferous MEAM1/B and MED/Q. Effects of ρ-cymene, thujene and neophytadiene on preference of whiteflies in Y-tube olfactometer were compared with general linear model (GLM). SPSS version 20.0 (SPSS Inc., Chicago, IL, United States) was used for all statistical analyses.

## Author Contributions

XS, XZ, and YZ designed the experiments. XS and GC performed the experiments. HP, WX, QW, SW, YL, XZ, and YZ contributed reagents and materials. XS and XZ wrote the paper.

## Conflict of Interest Statement

The authors declare that the research was conducted in the absence of any commercial or financial relationships that could be construed as a potential conflict of interest.
